# Deciphering microRNA-mRNA regulatory network in adult T-cell leukemia/lymphoma; the battle between oncogenes and anti-oncogenes

**DOI:** 10.1371/journal.pone.0247713

**Published:** 2021-02-25

**Authors:** Mohadeseh Zarei Ghobadi, Rahman Emamzadeh, Sayed-Hamidreza Mozhgani

**Affiliations:** 1 Faculty of Biological Science and Technology, Department of Cell and Molecular Biology and Microbiology, University of Isfahan, Isfahan, Iran; 2 Department of Microbiology, School of Medicine, Alborz University of Medical Sciences, Karaj, Iran; 3 Non-communicable Diseases Research Center, Alborz University of Medical Sciences, Karaj, Iran; Wayne State University, UNITED STATES

## Abstract

Adult T-cell leukemia/lymphoma (ATLL) is virus-caused cancer that originates from the infection by human T-cell leukemia virus type 1. ATLL dysregulates various biological pathways related to the viral infection and cancer progression through the dysexpression of miRNAs and mRNAs. In this study, the potential regulatory subnetworks were constructed aiming to shed light on the pathogenesis mechanism of ATLL. For this purpose, two mRNA and one miRNA expression datasets were firstly downloaded from the GEO database. Next, the differentially expressed genes and miRNAs (DEGs and DE-miRNAs, respectively), as well as differentially co-expressed gene pairs (DCGs), were determined. Afterward, common DEGs and DCGs targeted by experimentally validated DE-miRNAs were explored. The oncogenic and anti-oncogenic miRNA-mRNA regulatory subnetworks were then generated. The expression levels of four genes and two miRNAs were examined in the blood samples by qRT-PCR. The members of three oncogenic/anti-oncogenic subnetworks were generally enriched in immune, virus, and cancer-related pathways. Among them, *FZD6*, *THBS4*, *SIRT1*, *CPNE3*, miR-142-3p, and miR-451a were further validated by real-time PCR. The significant up-regulation of *FZD6*, *THBS4*, and miR-451a as well as down-regulation of *CPNE3*, *SIRT1*, and miR-142-3p were found in ATLL samples than normal samples. The identified oncogenic/anti-oncogenic subnetworks are pieces of the pathogenesis puzzle of ATLL. The ultimate winner is probably an oncogenic network that determines the final fate of the disease. The identified genes and miRNAs are proposed as novel prognostic biomarkers for ATLL.

## Introduction

Human T-cell leukemia virus type-1 (HTLV-1) is the cause of two main diseases including adult T-cell leukemia/lymphoma (ATLL) and HTLV-1-associated myelopathy/tropical spastic paraparesis (HAM/TSP). ATLL is a type of cancer that is recognized by the malignant lymphoproliferation and aggressive T-cell proliferation which are elevated in the infected cases after possibly a lengthy latency period [1]. It occurs in approximately 5% of individuals infected. The poor prognosis of ATLL is a substantial concern in the endemic region [2]. Two proteins including Tax and HTLV-1 basic leucine zipper factor (HBZ) are the main gene products of HTLV-1 which implicate the viral pathogenesis. HTLV-1 Tax is mostly detected at the mRNA level in the HAM/TSP patients. However, the HBZ mRNA level is more detectable in the PBMCs of ATLL patients [3]. The dysregulation of many other genes and proteins such as cytokines have been detected in ATLL cases [1]. Autophagy is a known homeostatic lysosome-employing process to degrade the nonspecific intracellular proteins in normal cells and also pathogens such as viruses [4]. The over-replication of HTLV-1 can be performed by the modulatory effect of the virus on the autophagy pathways [5].

MicroRNAs (miRNAs) are small non-coding RNAs including a length of nearly 19–25 nucleotides. They target various genes that involve in many biological processes such as cell cycle, proliferation, differentiation, apoptosis, and immune response [6,7]. The function of miRNAs as the post-transcriptional regulators can lead to degradation or translational suppression of their mRNA targets [8]. The possible roles of several miRNAs in HTLV-1 infection and ATLL progression especially in virus production, establishment of latency, and proliferation have been evaluated [9,10]. It has been proposed that the combination of miRNAs with chemotherapy drugs can be a proper treatment for ATLL due to overcoming chemo-resistance.

Nowadays, the high-throughput analysis provides the possibility of simultaneous determining thousands of genes. It may lead to finding the regulatory networks between different RNA types and identification of major players in progression of disease. The miRNA-mRNA regulatory network can shed light on the pathogenesis mechanism of diseases and also the identification of potential biomarkers and therapeutic agents.

Differential co-expression analysis (DCA) is a valuable approach in which changes in the co-expression patterns between the two conditions are calculated. DCA is complementary to the traditional differential expression analysis method since it considers the interaction between genes and finds the differential interacted genes due to a certain biological condition [11].

Herein, for the first time, we constructed the miRNA-mRNA regulatory subnetworks for the ATLL to identify potential regulators in the development of the pathogenesis process. To this end, we identified differentially expressed genes (DEGs) and differentially co-expressed gene pairs (DCGs). After determining the differentially expressed miRNAs (DE-miRNAs), we found the shared genes among DEGs, DCGs, and experimentally validated targets of DE-miRNAs. Next, we constructed the possible oncogenic and anti-oncogenic miRNA-mRNA regulatory subnetworks. Eventually, we validated four genes and two miRNAs in the networks.

## Materials and methods

### Datasets and preprocessing

Two mRNA expression datasets with accession numbers GSE33615 and GSE55851 as well as a miRNA expression dataset with accession number GSE31629 were downloaded from Gene Expression Omnibus (GEO) database (https://www.ncbi.nlm.nih.gov/geo/). They contain the expression profiles of normal and ATLL subjects (CD4+ cells). The details of the datasets are shown in Table 1. Two mRNA datasets were merged and their common genes were identified for further mRNA analysis. The merged mRNA and miRNA expression data were quantile normalized and log2 transformed. As some probe IDs were assigned to similar genes, the average expression was considered for repetitive genes.

**Table 1:** Characteristics of microarray datasets.

Accession number	Platform (GPL)	Samples
GSE31629	Agilent-019118 Human miRNA Microarray 2.0 G4470B (GPL7731)	ATLL: 40 Normal: 22
GSE55851	Agilent-014850 Whole Human Genome Microarray 4x44K G4112F (GPL4133)	ATLL: 12 Normal: 3
GSE33615	Agilent-026652 Whole Human Genome Microarray 4x44K v2 (GPL10332)	ATLL: 52 Normal: 21

### Identification of differentially expressed miRNAs (DE-miRNAs) and mRNA (DEGs)

To find DE-miRNAs and DEGs between ATLL and healthy samples, the R-based package of limma was applied. The false discovery rate (FDR) correction was computed using the Benjamini- Hochberg (BH) method (FDR < 0.05). The |logFC| > 1.5 and |logFC| > 2 were considered as the thresholds for identifying DE-miRNAs and DEGs, respectively.

### Detection of differentially co-expressed genes (DCGs)

To detect the differentially co-expressed genes, the diffcoexp package in R software was used. Employing this package, the differentially co-expressed links (DCLs) were initially identified. To this end, the Pearson correlation coefficients (PPCs) of all DEG pairs in ATLL and normal datasets were calculated and compared using Fisher’s Z-transformation.

The q.diffcor less than 0.005 was considered to determine the significantly differential co-expressed links. The gene pairs which had a remarkable discrepancy in PCC between two conditions and at least in one of them were considered as differentially co-expressed genes.

### Determination of target genes of DE-miRNAs

To determine the experimentally validated downstream target genes of DE-miRNAs, miRTarBase [12] database was employed.

### Pathway enrichment analysis

The common genes among DEGs, DCGs, and target genes of DE-miRNAs were enriched in KEGG and Reactome databases using g:Profiler (version: 1185_e69_eg16). The shared expressed genes between ATLL and healthy groups were considered as the background. The terms with adj.P.value < 0.05 were considered as statistically significant.

### Study participants and sample collection

The blood samples of 8 patients with ATLL and 10 normal individuals were collected with informed consent between 2019 and 2020 from the Shariati and Imam Khomeini Hospitals. Participants were informed about the study and then they agreed to participate and provided a written informed consent. A standardized clinical checklist, comprising demographic information and the diagnosis of ATLL was evaluated by a trained hematologist. The inclusion criteria for patients were subjects who did not treat with chemotherapy and anti-cancer drugs. All methods were carried out in accordance with the relevant guidelines and regulations. The enzyme-linked immunosorbent assay (ELISA, Diapro, Italy) was used to perform the seropositive test for HTLV-1. Polymerase chain reaction (PCR) was then employed to confirm the serology results [13]. This study was approved by the Ethics Committee of Biomedical Research at Alborz University of Medical Sciences (IR.ABZUMS.REC.1398.109).

### Quantitative real-time PCR

According to the manufacturer’s instructions, total RNA was extracted from fresh whole blood utilizing TriPure isolation reagent (Roche, Germany). cDNA was synthesized employing the RNJia PB Kit (ROJETechnologies, Iran). SYBR Green-based RT-qPCR was employed by SYBR Green (TaKaRa, Otsu, Japan), according to the manufacturer’s instructions. The relative 2 standard curves real-time PCR was performed on the cDNA samples using Q-6000 machine (Qiagen, Germany). The GAPDH gene and U6 miRNA were utilized as the housekeeping genes to normalize the mRNA and miRNAs expression levels respectively, as well as to control the error between samples [13,14]. The list of the designed primers and probes for determining the expression levels of SIRT1, FZD6, THBS4, CPNE3 genes and miRNAs including miR-451a [15,16] and miR-142 [17,18] are indicated in Table 2.

**Table 2:** List of designed primers to determine four genes.

Primers	Forward (5’→3’)	Reverse (5’→3’)
**SIRT1**	AGGAGCAGATTAGTAGGCGGCTT	AGCTCTCTCTGGAACATCAGGCT
**FZD6**	TGCTGTCTTCTGGGTTGGAAGC	GCTCCTGTGCTGGTTCCCAT
**THBS4**	CATGGTGCAGGGTGTTGGGA	GGCCCACAGCGGTAAGATCC
**CPNE3**	GGCTCCAATGGTGACCCAAGG	TGAGGAGGTATCTGAGCGCCAA
**miR-451a**	AAACCGTTACCATTACTGAGTT	GTGCAGGGTCCGAGGTATTC
**miR-142-3p**	CTCCTGTAGTGTTTCCTAC	GACTGTTCCTCTCTTCCTC
**U6**	CTCGCTTCGGCAGCACA	AACGCTTCACGAATTTGCGT

### Statistical analysis

Statistical analysis was carried out using GraphPad Prism Software Version 8.0.2 (GraphPad software, Inc). Quantitative data were expressed as mean ± SEM and percentages. The Mann Whitney t-test analysis was applied to compare two groups. The p ≤ 0.05 was considered as the significant difference.

## Results

### Identification of DE-miRNAs and DEGs

**Fig 1:**
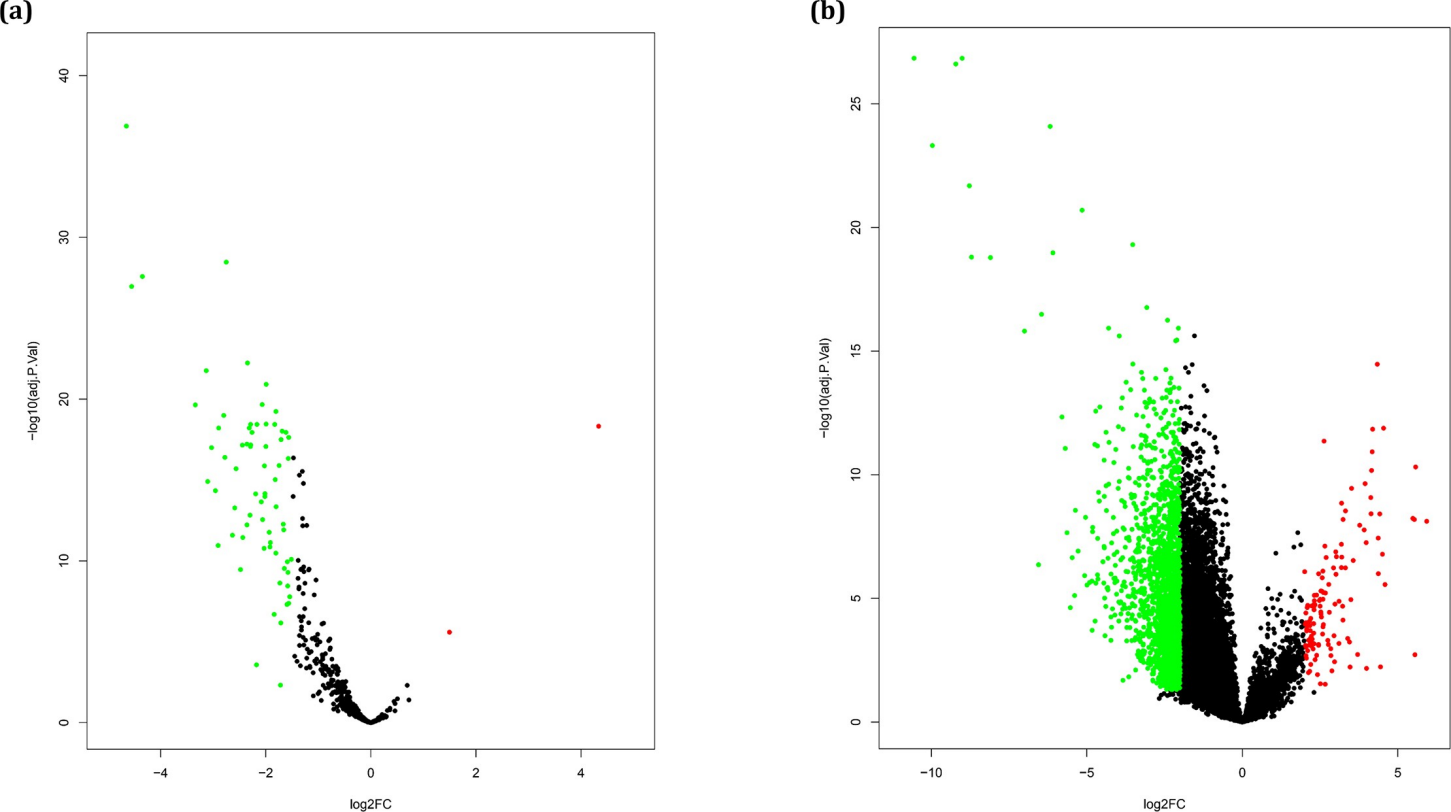
(a) Volcano plot of DE-miRNA and (b) DEGs between ATLL and healthy samples. Red and green dots display both significantly up-regulated and down-regulated DE-miRNA and DEGs in ATLL samples, respectively; black dots represent no significant difference.

A total of 16373 genes and 583 miRNAs were analyzed to find the differentially expressed miRNAs and genes, respectively. The analyses led to the identification of 70 DE-miRNAs and 2144 DEGs, respectively (S1 Table). Among the DE-miRNAs, 1 and 69 miRNAs were significantly up-regulated and down-regulated in ATLL samples, respectively (Fig 1A). Moreover, a total of 2020 DEGs were down-regulated and 124 DEGs were up-regulated (Fig 1B).

### Prediction of target genes of DE-miRNAs

In order to predict the downstream target genes of DE-miRNAs, miRTarBase was explored. Among 70 DE-miRNAs, a total of 31 miRNAs were found that target 6321 experimentally validated downstream genes (10265 miRNAs-target gene interaction, S2 Table).

### Detection of DCGs

Through the identification of differential co-expression genes, significant information regarding the changes in biological systems in response to a biological perturbation can be obtained [19]. Therefore, we utilized diffcoexp package to determine DCGs. Aa a result, a total of 77921 differentially co-expressed links were found (S3 Table, Sheet 1). A total of 90 significant differentially co-expressed were identified (S3 Table, Sheet 2). All DCGs were also among the shared DEGs. Therefore, they were considered for future analysis as a result of two analyses including differentially expressed and co-expressed genes. The DCGs were targeted by 28 down-regulated miRNAs and only one up-regulated miRNA (S3 Table, Sheet 3). These genes and miRNAs have biological functions related to infection and cancer development such as regulation of cell differentiation and proliferation, regulation of programmed cell death, cytokine-mediated signaling pathway, and regulation of apoptotic process.

### Pathway enrichments analysis

**Fig 2:**
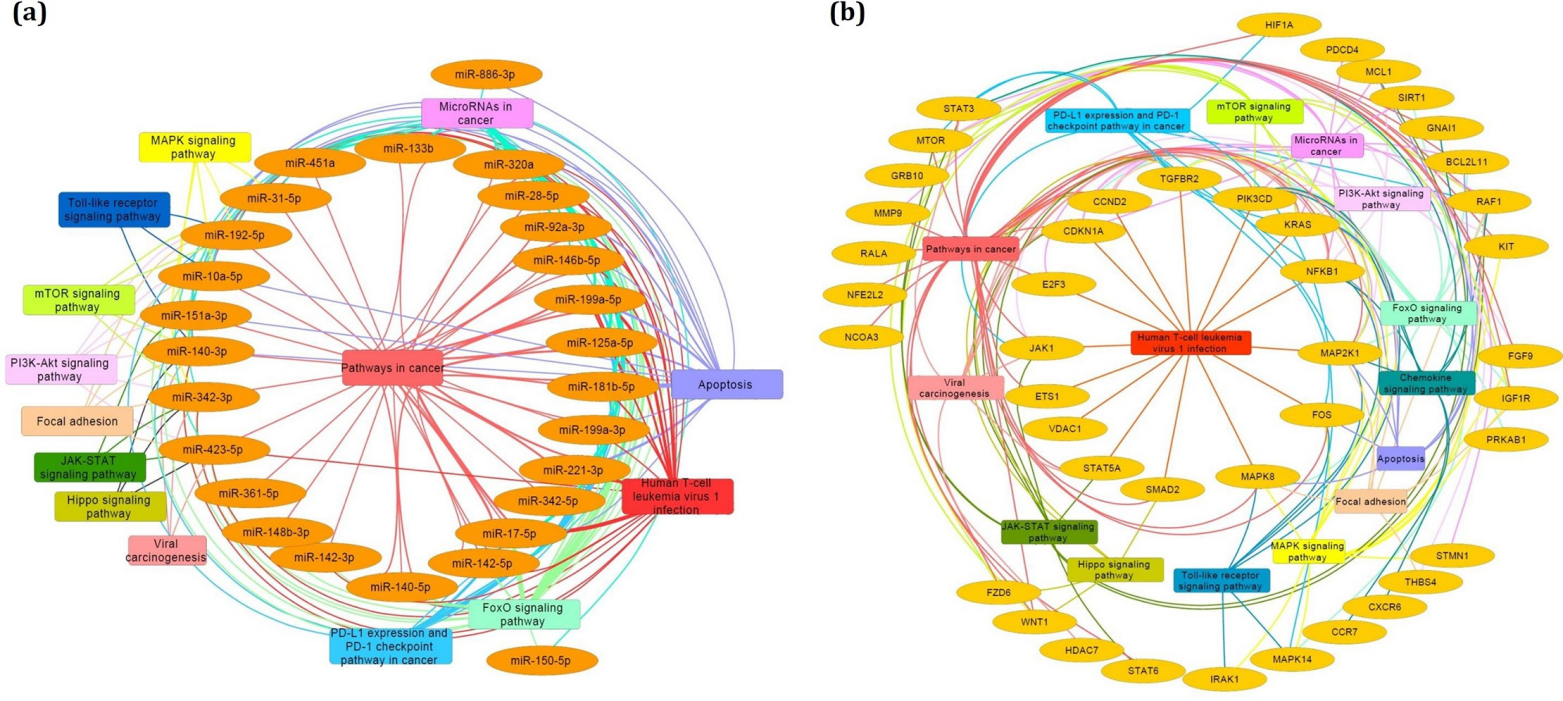
Pathway enrichment analysis of (a) DE-miRNAs and (b) DCGs. The edge colors correspond to each pathway and indicate the genes and miRNAs that enriched in them.

To find the pathways enriched by DE-miRNAs and DCGs, the KEGG and Reactome pathway enrichment analyses were carried out. Fig 2A and 2B represents the activation of genes and miRNAs in the cancer-, viral infection-, and immune-related pathways including Pathways in cancer, FoxO signaling pathway, MicroRNAs in cancer, Human T-cell leukemia virus 1 infection, PD-L1 expression and PD-1 checkpoint pathway in cancer, MAPK signaling pathway, PI3K-Akt signaling pathway, JAK-STAT signaling pathway, Apoptosis, mTOR signaling pathway, Toll-like receptor signaling pathway, Chemokine signaling pathway, Viral carcinogenesis, Focal adhesion, and Hippo signaling pathway. It discloses the integration of virus and cancerous factors to progress the malignant ATLL.

### Construction of miRNA-mRNA regulatory subnetwork

**Fig 3:**
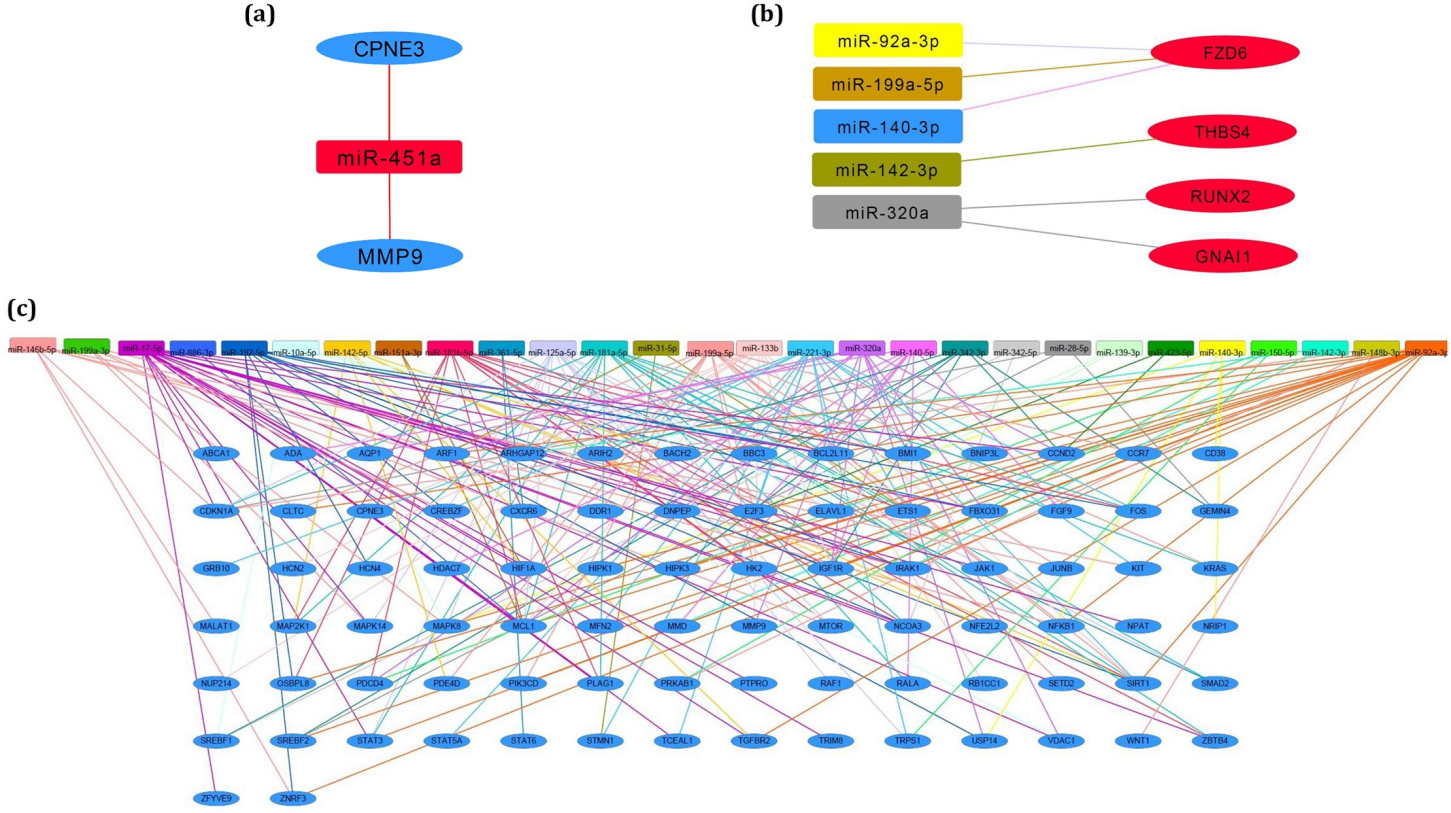
The miRNA-mRNA regulatory subnetworks between (a) up-regulated miRNAs and down-regulated DCGs, (b) down-regulated miRNAs and up-regulated DCGs, (c) down-regulated miRNAs and down-regulated DEGs. The edge colors correspond to each miRNA and display the genes that interacted with them. The red and blue gene colors represent the up-regulated and down-regulated DCGs, respectively.

The identified DE-miRNAs and mRNAs (DEGs and DCGs) constitute a large network containing oncogenic and anti-oncogenic parts. To clarify miRNA-mRNA relationships, three miRNA-mRNA regulatory subnetworks were constructed. Fig 3A shows the anti-oncogenic subnetwork generated between up-regulated miR-451a and down-regulated DCGs including CPNE3 and MMP-9 (S4 Table, sheet 1). This subnetwork implicates the regulation of cell migration, neutrophil activation involved in immune response, response to growth factor, and regulation of apoptotic signaling pathway. An oncogenic/anti-oncogenic subnetwork containing five interactions was found between down-regulated DE-miRNAs including miR-92a-3p, miR-140-5p, miR-199a-5p, miR-320a, miR-142-3p and up-regulated DCGs containing *FZD6*, *RUNX2*, *THBS4*, and *GNAI1* (Fig 3B, S4 Table, sheet 2). The gene members of this subnetwork have biological functions in the regulation of cAMP-mediated signaling, regulation of endothelial cell proliferation, regulation of cell differentiation, and regulation of angiogenesis. In order to construct the regulatory subnetwork between down-regulated DCGs and down-regulated DE-miRNAs, another oncogenic/anti-oncogenic network was constructed (Fig 3C). The subnetwork contains interactions between various miRNAs and mRNAs (S4 Table, sheet 3). This subnetwork implicates many biological processes such as regulation of cell migration and proliferation, cellular response to cytokine stimulus, regulation of apoptotic process, regulation of JAK-STAT cascade, regulation of growth, cytokine-mediated signaling pathway, and inflammatory response.

### Demographic and clinical data of studied subjects

This study surveyed 8 seropositive acute ATLL patients (3 women and 5 men; mean age 53.34 ± 5.94 years, respectively) and 10 normal adult donors (4 women and 6 men; mean age 48.92 ± 5.86 years, respectively). There was no significant difference between age in the ATLL and normal subjects (p = 0.12). Among the patients, 5 patients had lymphadenopathy, 2 patients had immunodeficiency disorders as well as skin lesion. One patient had lymphadenopathy and skin lesion simultaneously. None of the patients showed three clinical symptoms, simultaneously.

### Real time-quantitative PCR for a subset of genes and miRNAs

**Fig 4:**
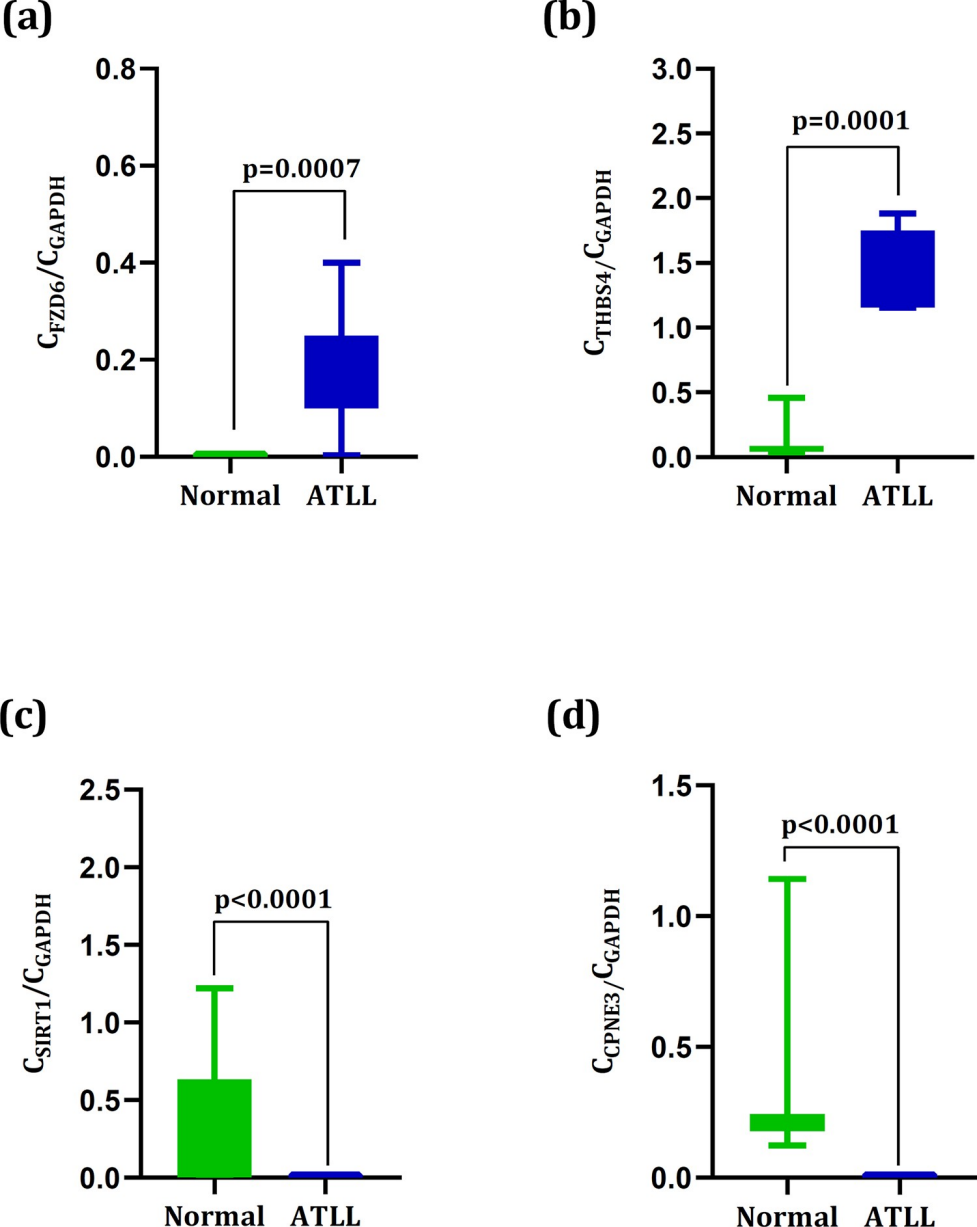
The expression levels of (a) *FZD6*, (b) *THBS4*, (c) *SIRT1*, and (d) *CPNE3* in the normal and ATLL groups. The expression levels of *FZD6* and *THBS4* in the ATLL samples were significantly higher than in normal (p< 0.0001 and p< 0.0001). The expression levels of *SIRT1* and *CPNE3* in the ATLL samples were significantly lower than in normal (p = 0.0007 and p = 0.0001).

**Fig 5:**
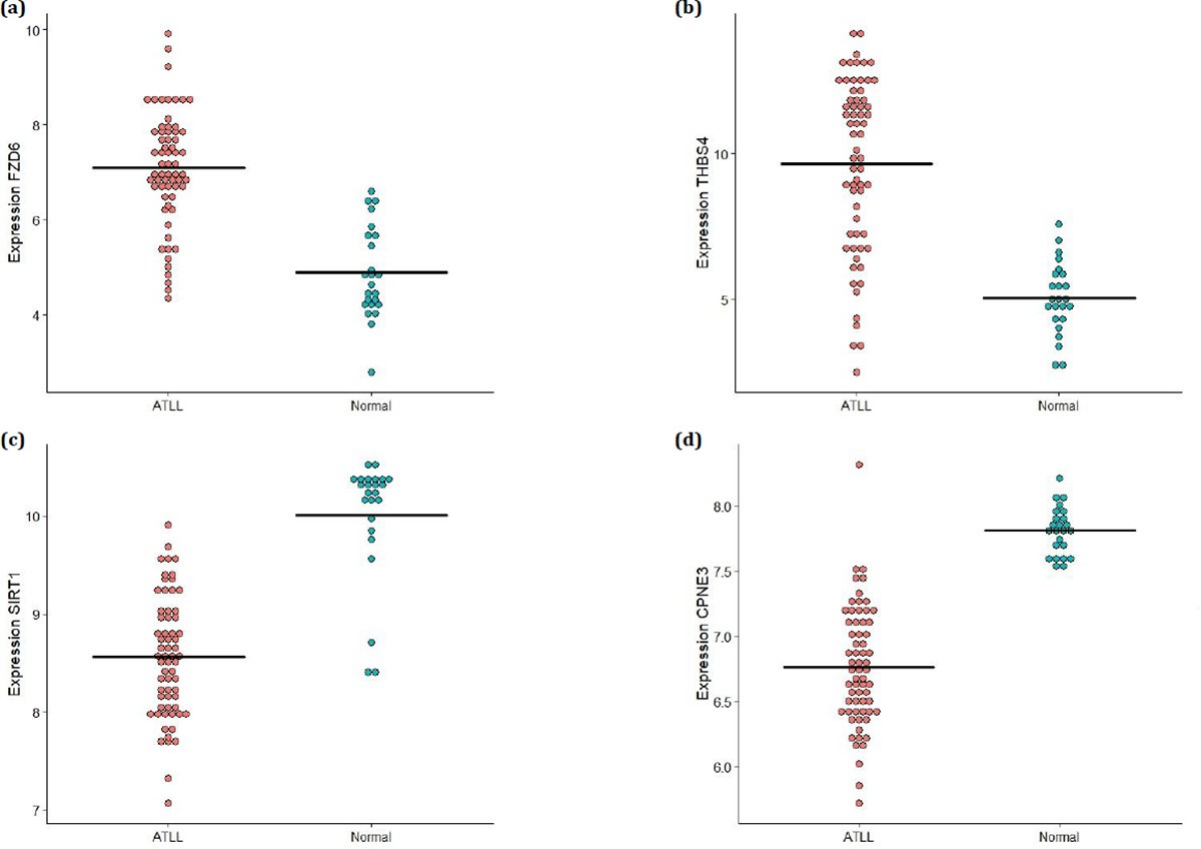
The expression levels of (a) *FZD6*, (b) *THBS4*, (c) *SIRT1*, and (d) *CPNE3* in the microarray datasets.

**Fig 6:**
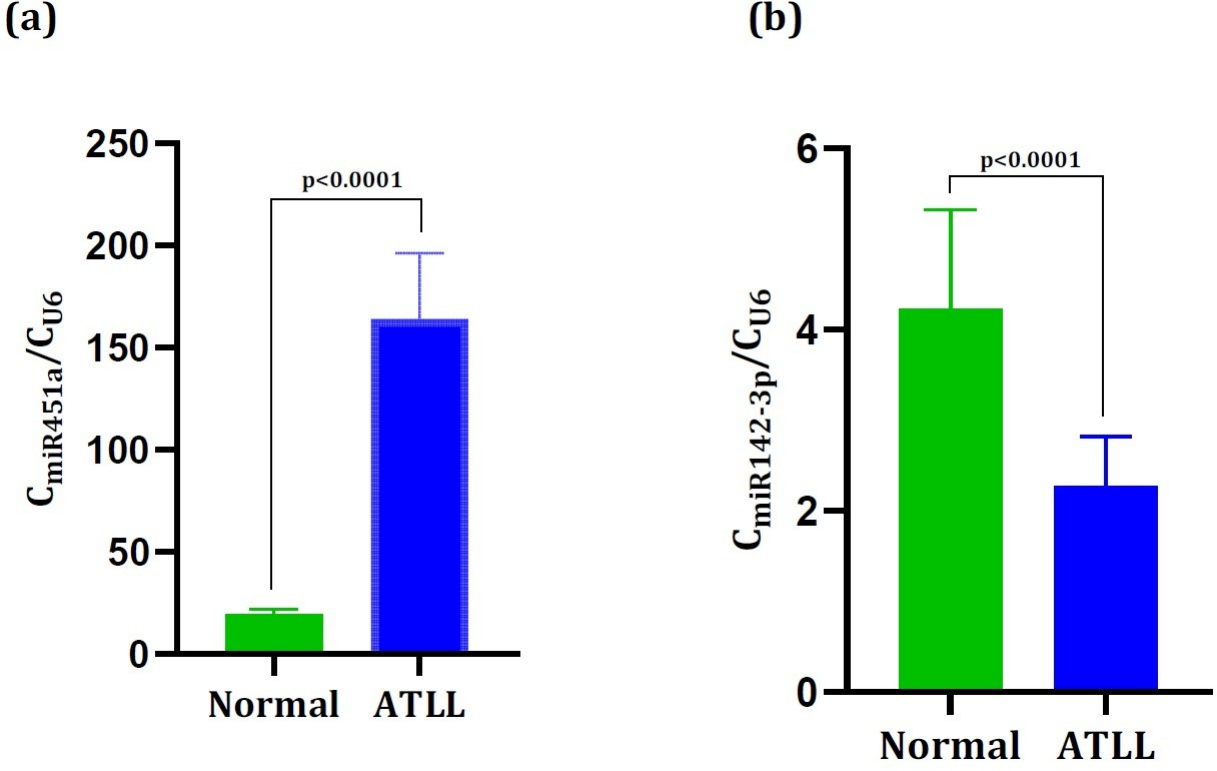
The expression levels of (a) miR-451a and (b) miR-142-3p in the normal and ATLL groups. The expression levels of miR-451a and miR-142-3p in the ATLL samples were significantly higher and lower than those in normal, respectively (p< 0.0001).

A total of four genes including *FZD6*, *THBS4*, *SIRT1*, and *CPNE3* were chosen to be evaluated by real time-quantitative PCR analysis. These genes were found as DEGs and also as DCGs which were targeted by up-regulated and down-regulated miRNAs. The results showed the remarkable up-regulation of *FZD6* (0.17± 0.04) and *THBS4* (1.48± 0.10) in ATLL samples than those in the normal samples (0.0018± 0.0005) and (0.10 ± 0.04) with *p =* 0.0007 and *p* = 0.0001, respectively (Fig 4A and 4B). Also, the significant down-regulation of *SIRT1* (2.6×10^−5^± 6.7×10^−6^) and *CPNE3* (0.0035± 0.0001) were observed in ATLL samples than those in the normal samples (*SIRT1*: 0.29± 0.13) and (*CPNE3*:0.28± 0.09), all with *p*< 0.0001 (Fig 4C and 4D). The results were also consistent with their expression values in the microarray datasets (Fig 5A–5D). Moreover, we validated miR-142-3p and miR-451a. The outcomes revealed the significant up-regulation of miR-451a (19.5± 0.785) and down-regulation of miR-142-3p (2.26± 0.196) in ATLL samples than those in the normal samples (miR-142-3p: 4.22± 0.346 and miR-451a: 164± 11.4), all with *p*< 0.0001 (Fig 6A and 6B).

## Discussion

The considerable linkage between miRNA-mRNA regulatory networks and the development of various cancers have been reported in several reports [20–22]. However, such a study has not been yet performed regarding ATLL as pathogen-caused cancer. In the present study, a total of 29 miRNAs and 90 genes were identified which have been determined as differentially expressed in ATLL samples. Among them, 1 miRNA and 4 genes were up-regulated and 28 miRNAs and 86 genes were down-regulated. All DEGs were also determined as differentially co-expressed genes. Pathway enrichment analysis unveiled the enrichment of miRNAs and mRNAs in cancer and virus-caused pathways. In order to survey the oncogenic/anti-oncogenes functions of miRNA-mRNA subnetworks, they were separately studied. In the anti-oncogenic subnetwork, the down-regulated *MMP-9* targeted by up-regulated miR-451a were enriched in Pathways in cancer. Different expression behaviors have been reported for miR-451a since it down-regulated in melanoma [23], acute myeloid leukemia [24], and lung adenocarcinoma [25]. Conversely, it was up-regulated in pancreatic cancer [26] and Adrenal Myelolipoma [27]. The increase in the expression level of miR-451a is possibly due to a positive feedback system and the progression of ATLL [28]. The miR-451a directly targets *MMP-9* and *CPNE3* and may negatively regulate their expressions in ATLL patients. *MMP-9* is a tumor-derived matrix metalloproteinase that has a key role in tumor progression, metastasis, and tumor-induced angiogenesis [29]. The overexpression of *MMP-9* in the ATLL patients may occur through the activation of *MMP-9* promoter by the mediation of viral oncoprotein Tax [30,31]. However, the genetic and epigenetic alterations such as deletion of 5′‐LTR, nonsense mutation, and DNA methylation of 5′‐LTR lead to the loss of Tax protein and escape from the host immune system [32]. Therefore, the down-regulation of *MMP-9* in the ATLL is expected. *CPNE3* is another target gene that is down-regulated due to the possible function of miR-451a. *CPNE3* encodes a Ca^2+^-dependent phospholipid-binding protein termed copine III. It promotes tumor progression and migration through interaction with ErbB2 [33]. The higher expression of *CPNE3* has been observed in several cancers, however, the decrease in its expression level was also observed in prostate cancer [34]. Therefore, it should be investigated in further studies in terms of genetic and epigenetic changes. *MMP-9* and *CPNE3* were also targeted by miR-133b which was down-regulated in ATLL. It is in consistent with other cancers like bladder, non-small cell lung, colorectal, head and neck/oral, and esophageal squamous cell cancer [35]. MiR-133b prevents cell migration and invasion in cancers.

In the oncogenic subnetwork, the overexpression of *FZD6* targeted by miR-140-5p, miR-92a-3p, and miR-199a-5p as the known tumor suppressors were observed in ATLL. They were enriched in Pathways in cancer and Proteoglycans in cancer. The negative regulation of *FZD6* expression by miR-199a-5p has been observed in colorectal cancer [36]. *FZD6* belongs to the ’frizzled’ gene family, which encodes protein receptors for Wnt signaling proteins. FZD6 likely activates the planar cell polarity pathway by directing cell migration during organogenesis [37]. The up-regulation of *FZD6* coupled with down-regulation of the transcription factor *TCF4* was previously proposed to predict the low survival of glioblastoma patients [38]. Similarly, *FZD6* can be proposed as the prognostic biomarkers in ATLL patients. MiR-320a is another DE-miRNA in the oncogenic subnetwork which acts as a tumor suppressor and apoptosis-inducing agent [39]. The down-regulation of miR-320a is accompanied by the up-regulation of *RUNX2* and *GNAI1*. RUNX2 is a transcription factor that has a key function in osteoblastic differentiation and skeletal morphogenesis. It is also known as a proto-oncogene and a pro-metastatic factor [40]. Moreover, RUNX2 reinforces autophagy through α-tubulin acetylation and autophagic vesicle trafficking [41]. Therefore, it can elevate T-cell survival and simplify malignant transformation in ATLL through promoting autophagy. GNAI1 belongs to the Gα inhibitory family which implicates various biological processes such as adhesion, proliferation, and differentiation [42]. The overexpression of GNAI1 in ATLL samples makes it as a prognostic biomarker candidate.

Another member of the oncogenic subnetwork is *THBS4*. It pertains to a calcium-binding glycoprotein family and has key roles in regulating cell–cell and cell–matrix interactions, synaptogenesis, and angiogenesis [43,44]. THBS4 is expressed by cancer-associated fibroblasts. It can be triggered by the tumor cells [45]. In this study, the miR-142-3p was down-regulated which may lead to the up-regulation of *THBS4*, Therefore, it may promote tumorigenesis, cell migration, and invasion [46] in ATLL.

Although miRNAs have a negative regulatory effect on their mRNA targets, each mRNA is targeted by several miRNAs. Therefore, a complex network comprising oncogenic/anti-oncogenic parts involve in the progression of the disease. Therefore, we also explored the down-regulated mRNAs and miRNAs subnetwork.

*SIRT1* is a gene member of the mentioned oncogenic/anti-oncogenic subnetwork whose down-regulation is contrary to the previous study. However, two contradictory roles have been considered for *SIRT1* including tumor promoter and tumor suppressor. The down-regulation of *SIRT1* leads to tumorigenesis and up-regulation of *SIRT1* inhibits the activity of several oncogenes such as *HIC1* and *DBC1* resulting in cell proliferation, apoptosis, and tumor suppression [47]. A decrease in the expression level of *SIRT1* in ATLL can enhance the process of tumorigenesis.

Taken together, a complicated network involves the ATLL pathogenesis in which the expression of genes is regulated by several up- or/and down-regulated miRNAs. Therefore, the down-regulated miRNA/down-regulated mRNA is part of a complex network. The network constitutes from the oncogenic and anti-oncogenic subnetworks of miRNA and mRNA. It is speculated that the final conqueror is an oncogenic network that determines the final destiny of disease. However, further study on a larger cohort should be carried out to validate the discriminative ability of the identified biomarkers and to explore their biological communications [48].

## Conclusion

In conclusion, we constructed the potential oncogenic/anti-oncogenic miRNA-mRNA regulatory subnetworks that may be involved in the development of ATLL. FZD6, THBS4, SIRT1, CPNE3, miR-142-3p, and miR-451a are the members of the regulatory network which we confirmed their expression levels. The introduced miRNAs and mRNAs may propose as the potential prognosis biomarkers and also therapeutics factors. However, due to targeting multiple genes by various dysregulated miRNAs, further studies should be designed and performed to evaluate the complex networks between miRNA and genes.

## Supporting information

S1 Table List of differentially expressed miRNAs and mRNAs. (XLSX)

S2 Table List of common miRNAs between DE-miRNAs and miRTarBase and their target genes. (XLSX)

S3 Table List of DCLs (sheet 1), DCGs (sheet 2), miRNA-DCGs (sheet 3). The downregulated miRNAs are specified by blue color and up-regulated miRNAs are specified by red color.
(XLSX)

S4 Table List of miRNAs and mRNAs in up-regulated DE-miRNAs/down-regulated DCGs (sheet 1), down-regulated DE-miRNAs/up-regulated DCGs (sheet 2), and down-regulated DE-miRNAs/down-regulated DCGs (sheet 3). (XLSX)
